# Malaria Eliminating: What Lies Ahead of Iran?

**Published:** 2018-03-18

**Authors:** Hamed Ebrahimzadeh-Leylabadlo

**Affiliations:** Infectious and Tropical Diseases Research Center, Faculty of Medicine, Tabriz University of Medical Sciences, Tabriz, Iran

## Dear Editor-in-Chief

In recent years, Iran has made significant advances on the fight against malaria with a stated objective of complete elimination. According to the report of Iranian Ministry of Health and Medical Education, the annual number of malaria cases have been reduced from 12294 in 2000 to 787 in 2012, 366 cases in 2014 and only 150 cases reported in 2015. In 2009, WHO malaria report, announced Iran was in the pre-elimination phase (1).

Malaria incidence had significant reduction during last years, however, Iran is facing a number of operational problems in a fight against Malaria. Around 90% of the locally transmitted malaria cases happen over the southeastern endemic regions in Iran every year (2). The imported malaria cases and cross-border movement of population from malaria-endemic countries particularly Pakistan and Afghanistan that enter to Sistan and Baluchestan and Khorasan-Razavi Provinces illegally via the eastern Iranian borders. The burden of most infectious diseases, including malaria, leishmaniasis, and tuberculosis in Afghanistan is more noteworthy than in Iran and individuals Afghani are most of the migrants in Iran (3).

On the other hand, global climatic change is expected to increase the incidence of vector-borne diseases, especially malaria. Many countries in the world including Iran have been hit by climate change and diseases like malaria and leishmaniasis may change pattern and appear in provinces not prevalent before (4). Thus, despite the reduction of malaria cases in recent years, the risk of reintroduction is high and need to be continued cross-border collaborations with endemic neighbors to pursuing the goal of completely malaria elimination by 2020.
